# Tripartite exacerbation stratification in AECOPD suggests a gradient of lower airway dysbiosis: a metagenomic transition from commensal taxa to *pseudomonadota* dominance

**DOI:** 10.3389/fmicb.2025.1588029

**Published:** 2025-11-24

**Authors:** Yunxia An, Min Xu, Yi Kang, Jinzhou Fang, Xiaoju Zhang

**Affiliations:** 1 Department of Respiratory and Critical Care Medicine, Zhengzhou University People's Hospital, Henan Provincial People's Hospital, Zhengzhou, China; 2 Department of Infectious Diseases, Zhengzhou University People's Hospital, Henan Provincial People's Hospital, Zhengzhou, China

**Keywords:** chronic obstructive pulmonary disease (COPD), lung microbiome, bronchoalveolar lavage fluid (BALF), exacerbation frequency, metagenomic next-generation sequencing (mNGS)

## Abstract

**Background:**

The frequency of acute exacerbations (AECOPD) is a critical predictor of disease progression in chronic obstructive pulmonary disease (COPD). However, the dynamics of the lower respiratory microbiome across a spectrum of exacerbation frequency remain poorly characterized, limiting insights into microbial drivers of susceptibility.

**Methods:**

We conducted a cross-sectional study of 39 hospitalized AECOPD patients, stratified into non-frequent (NFE, ≤ 1 event/year, *n* = 11), moderate (ME, 2 events/year, *n* = 13), and frequent exacerbators (FE, ≥3 events/year, *n* = 15). Metagenomic next-generation sequencing (mNGS) was performed on bronchoalveolar lavage fluid (BALF) to profile the airway microbiome.

**Results:**

Microbial alpha diversity exhibited a significant, graded decline from NFE to FE groups (e.g., Shannon index: NFE 3.68 ± 0.34, ME 3.02 ± 1.02, FE 0.84 ± 0.54; *p* < 0.05). Beta diversity analysis revealed distinct community clustering by exacerbation phenotype (PERMANOVA R^2^ = 0.19, *p* = 0.001). The FE group was characterized by a striking dominance of *Pseudomonadota* (relative abundance: 72.25%), which correlated positively with exacerbation frequency (*r* = 0.536, *p* < 0.001). In contrast, commensal taxa including *Streptococcus* (*r* = −0.814, *p* < 0.0001) and others within the *Bacillota* and *Bacteroidota* phyla were depleted in FE and were negatively associated with exacerbation frequency. Twelve exacerbation-resilient taxa (83.3% belonging to *Bacillota*/*Bacteroidota*) were positively correlated with FEV_1_% predicted (*r* = 0.322–0.483, *p* < 0.05). Alpha diversity indices showed a strong inverse association with exacerbation frequency (*r* = −0.84 to −0.86, *p* < 0.001) but not spirometric measures.

**Conclusion:**

Our findings delineate a gradient of airway microbial dysbiosis along the exacerbation frequency spectrum in COPD. The exacerbation-prone phenotype is defined by a loss of microbial diversity, expansion of *Pseudomonadota*, and depletion of potentially protective commensals. These microbiome features represent promising biomarkers for identifying high-risk patients and may inform future microbiome-targeted therapeutic strategies.

## 1 Introduction

Chronic obstructive pulmonary disease (COPD), the third leading cause of global mortality, accounts for over 3 million annual deaths and imposes substantial healthcare burdens through progressive lung function deterioration and recurrent acute exacerbations (AECOPD) (World Health Organization, 2020; Rabe et al., 2007; Wedzicha and Seemungal, 2007; GBD 2019 Diseases Injuries Collaborators, 2020). Although AECOPD frequency serves as a critical prognostic indicator of disease severity (Hurst et al., 2010), current therapeutic approaches remain insufficiently tailored to individual exacerbation risk profiles, reflecting gaps in understanding the microbial determinants of exacerbation-prone phenotypes (Han et al., 2017).

The airway microbiome has emerged as a central player in COPD pathogenesis, being intricately involved in inflammatory cascades and immune dysregulation (Dickson et al., 2014; Sethi and Murphy, 2008). However, traditional diagnostic tools—including culture-based methods and 16S rRNA gene sequencing—fail to capture the functional complexity of respiratory microbiota due to inherent taxonomic biases and limited resolution (Dickson et al., 2013; Biesbroek et al., 2012; Zhang et al., 2020). Recent advances in bronchoalveolar lavage fluid (BALF)-based metagenomic next-generation sequencing (mNGS) now enable high-resolution profiling of the lower airway microbiome, overcoming contamination issues associated with sputum analysis while providing strain-level taxonomic and functional insights (Gu et al., 2019).

Despite these technological breakthroughs, fundamental questions persist. Previous investigations have predominantly dichotomized COPD cohorts into frequent versus non-frequent exacerbators (Reilev et al., 2017; Le Rouzic et al., 2018), a binary classification that risks masking transitional microbial states during disease progression. We propose that a tripartite stratification strategy—categorizing patients by annual exacerbation frequency into ≤ 1 (non-frequent), 2 (moderate), and ≥3 (frequent) events—will uncover a microbial ecological continuum. Specifically, we hypothesize that frequent exacerbators exhibit progressive pathogen dominance (e.g., *Pseudomonadota phylum*) accompanied by collapse of alpha diversity, whereas non-frequent exacerbators retain protective commensal taxa (e.g., Streptococcus, Prevotella) associated with lung function preservation. Furthermore, we anticipate that microbiome-driven exacerbation risk operates independently of conventional spirometric indices, suggesting novel pathways for therapeutic targeting.

By integrating BALF mNGS with tripartite phenotyping, this study aims to delineate dynamic microbiome shifts along the AECOPD severity spectrum, thereby providing mechanistic insights into microbial drivers of exacerbation susceptibility and paving the way for personalized microbiome-modulating interventions.

## 2 Methods

### 2.1 Patient recruitment and grouping

This study was conducted in the Department of Respiratory Medicine at Henan Provincial People's Hospital from March 2021 to December 2023. We consecutively enrolled patients diagnosed with an acute exacerbation of chronic obstructive pulmonary disease (AECOPD) according to the 2023 Global Initiative for Chronic Obstructive Lung Disease (GOLD) criteria. The study protocol was approved by the Institutional Review Board of Henan Provincial People's Hospital, and written informed consent was obtained from all participants or their legally authorized representatives.

The inclusion criteria were: (1) worsening respiratory symptoms (cough, sputum production, dyspnea); (2) purulent or mucopurulent sputum; (3) post-bronchodilator ratio of forced expiratory volume in 1 s to forced vital capacity (FEV1/FVC) < 70%; and (4) no antibiotic use within 4 weeks prior to enrollment. Exclusion criteria included comorbidities such as heart failure, malignancy, autoimmune diseases, or contraindications to bronchoscopy.

Based on the frequency of acute exacerbations in the preceding year, patients were stratified into three groups: the frequent exacerbator (FE) group (≥3 episodes, *n* = 15), the moderate exacerbator (ME) group (2 episodes, *n* = 13), and the non-frequent exacerbator (NFE) group (≤1 episode, *n* = 11). Baseline demographic and clinical data, including gender, age, smoking history, alcohol consumption, comorbidities, mMRC score, and body mass index (BMI), were collected for all patients.

### 2.2 Bronchoalveolar lavage fluid collection and DNA extraction

Bronchoscopy and bronchoalveolar lavage fluid (BALF) collection were performed following a standardized protocol. The sampling site was determined by reviewing chest CT scans prior to the procedure: for localized lesions, the most severely affected subsegment was chosen; for diffuse lung disease, the right middle lobe or lingular segment of the left upper lobe was selected. After wedging the bronchoscope into the target bronchus, pre-warmed (37 °C) sterile saline was instilled in 20–50 mL aliquots to a total volume of 120 mL. The fluid was immediately aspirated under appropriate negative pressure (80–120 mmHg). The total fluid recovery rate was >40% for all analyzed samples. Qualified samples met the following cytological criteria: squamous epithelial cells <5% and red blood cells <10%. Samples were stored at −80 °C until DNA extraction.

Total DNA was extracted from BALF using the QIAamp UCP Pathogen DNA Kit (Qiagen, Hilden, Germany) according to the manufacturer's instructions. To reduce host DNA contamination, samples were pretreated with Benzonase (Qiagen) and 0.1% Tween-20 (Sigma-Aldrich, St. Louis, MO, USA) prior to extraction. DNA concentration and purity were assessed using a NanoDrop 2000 spectrophotometer (Thermo Fisher Scientific) and the Qubit dsDNA HS Assay Kit (Thermo Fisher Scientific).

### 2.3 Library preparation and metagenomic sequencing

Libraries were constructed using the Nextera XT DNA Library Prep Kit (Illumina) with 10 ng of high-quality genomic DNA as input. The quality of the resulting libraries was assessed using the Agilent 2100 Bioanalyzer with the High Sensitivity DNA Kit (Agilent Technologies, Santa Clara, CA, USA). Qualified libraries were pooled in equimolar concentrations and sequenced on the Illumina NextSeq 550Dx platform using a 75-cycle single-end strategy (NextSeq 500/550 High Output Kit v2.5), aiming to generate approximately 20 million raw reads per library. Peripheral blood mononuclear cells (PBMCs, 10^5^ cells/mL) from healthy donors served as a negative process control, and DNA-free water subjected to the entire DNA extraction and sequencing workflow served as a no-template control (NTC) to assess background contamination.

### 2.4 Bioinformatics analysis

Raw sequencing reads were quality-filtered using Trimmomatic v0.39 (Bolger et al., 2014) with the following parameters: SLIDINGWINDOW:4:20, MINLEN:50. Adapter sequences were removed using the Illumina adapter database. Host DNA contamination was minimized by aligning reads to the GRCh38 human reference genome using Bowtie2 v2.5.4 (Langmead and Salzberg, 2012) (very-sensitive-local mode). Taxonomic profiling was performed using Kraken2 v2.1.3 and Bracken v2.9 with the Standard Plus Protozoa & Fungi database (version 2024Q3).

Alpha diversity indices and Beta diversity were calculated using the Vegan package (v2.6.8) in R. Beta diversity, based on Bray-Curtis dissimilarity (calculated using the vegdist function), was used to assess differences in microbial community structure among groups via Permutational Multivariate Analysis of Variance (PERMANOVA) with 999 permutations. Visualization of beta diversity patterns was performed using Principal Coordinate Analysis (PCoA) (cmdscale()). The Linear Discriminant Analysis Effect Size (LEfSe) (Segata et al., 2011) method was employed to identify differentially abundant taxonomic features across groups, with a significance threshold set at an LDA score >3.5. All analyses were implemented in R v4.3.1.

### 2.5 Statistical analysis

Statistical analyses were performed using SPSS Statistics 21 (IBM, Armonk, NY, USA) and R software (v4.3.1). Normally distributed continuous variables are presented as mean ± standard deviation and were compared using one-way ANOVA. Non-normally distributed continuous variables are presented as median (interquartile range) and were compared using the Kruskal–Wallis test. Categorical variables are expressed as counts (percentages) and were compared using the Chi-square test or Fisher's exact test, as appropriate. The relative abundances of the top 10 microbial phyla, genera, and species were arcsine square root-transformed to normalize skewed distributions (Li et al., 2019), and group differences were assessed using the Kruskal–Wallis test. The Jonckheere–Terpstra trend test was used to analyze trends in microbial relative abundance across exacerbation frequency groups. Spearman's rank correlation was used to assess associations between bacterial relative abundance and clinical indicators. All statistical tests were two-sided, and a *p*-value < 0.05 was considered statistically significant.

## 3 Results

### 3.1 Patient characteristics

The study enrolled 39 AECOPD patients stratified by exacerbation frequency into: NFE (*n* = 11), ME (*n* = 13), and FE (*n* = 15) groups. Clinical characteristics, including gender, age, smoking history, BMI, mMRC scores, comorbidities, and inflammatory markers (NLR, CRP), showed no significant differences across groups (all *p* > 0.05; Table 1). However, the FE group exhibited significantly lower lung function compared to the NFE group, as evidenced by FEV_1_% predicted (34(20.0) vs. 62(30.9), *p* = 0.018) and FEV_1_/FVC% (41.68 ± 12.74 vs. 54.30 ± 10.9, *p* = 0.049) (Table 1).

**Table 1:** Clinical characteristics of the study population.

**Characteristics**	**NFE**	**ME**	**FE**	***P*-value**
*N*	11	13	15	
Age (years), median (IQR)	70(9)	68(13)	66(20)	0.562^$^
Sex, male, *n* (%)	8(72.73)	12(92.31)	12(80.00)	0.509^#^
BMI (kg/m^2^), mean ± SD	20.87 ± 2.34	22.95 ± 3.17	22.27 ± 3.64	0.279^*^
Smoking index (pack-years), median (IQR)	30(50)	30(45)	15(32)	0.584^$^
Drinking history				0.711^#^
Ever-drinker, *n* (%)	4(36.36)	4(30.77)	7(46.67)	
Never-drinker, *n* (%)	7(63.64)	9(69.23)	8(53.33)	
**Comorbidities**				
Hypertension (%), *n*	4(36.36)	5(38.46)	4(26.67)	0.762^#^
Diabetes mellitus (%), *n*	3(27.27)	1(7.69)	5(33.33)	0.278^#^
mMRC Score, median (IQR)	1(1)	2(1)	2(1)	0.834^$^
WBC (^*^10^9^/L), mean ± SD	7.40 ± 2.79	10.23 ± 4.59	7.89 ± 2.76	0.107^*^
NEUT (^*^10^9^/L), mean ± SD	5.09 ± 2.72	7.97 ± 4.56	6.21 ± 2.82	0.133^*^
NEUT (%), mean ± SD	66.52 ± 13.13	73.67 ± 12.90	76.92 ± 13.86	0.156^*^
LYM (^*^10^9^/L), median (IQR)	1.27(1.55)	1.27(0.89)	0.79(0.75)	0.107^$^
NLR, median (IQR)	2.26(4.23)	6.09(7.74)	6.56(7.71)	0.130^$^
Eos (^*^10^9^/L), median (IQR)	0.08(0.11)	0.11(0.24)	0.03(0.20)	0.674^$^
CRP (mg/L), median (IQR)	5.41(90.67)	51.57(48.31)	38.51(121.98)	0.165^$^
FEV_1_/pred (%), median (IQR)	62(30.9)	47(37.5)	34(20.0)	0.022^$^
FEV_1_/FVC, mean ± SD	54.30 ± 10.90	48.42 ± 13.78	41.68 ± 12.74	0.052^*^
History of COPD, median (IQR)	1(3)	7(10)	10(17)	0.053^$^

Continuous variables are presented as median (interquartile range, IQR) or mean ± standard deviation (SD); categorical variables are expressed as n (%). Differences among the three groups were assessed using one-way ANOVA (for normally distributed data) (^*^), Kruskal–Wallis test (for non-normally distributed data) ($), or Fisher's exact test (for categorical data) (#). Post-hoc analyses were performed with Bonferroni correction (for ANOVA) or Dunn's test (for Kruskal–Wallis test) when significant differences were found.

BMI, body mass index; mMRC, modified Medical Research Council; WBC, white blood cell; Neut, neutrophil; Lym, lymphocyte; Eos, eosinophil; NLR, neutrophil-to-lymphocyte ratio; CRP, C-reactive protein; FEV1, forced expiratory volume in 1 second; FVC, forced vital capacity; COPD, chronic obstructive pulmonary disease.

### 3.2 BALF microbial composition

**Figure 1:**
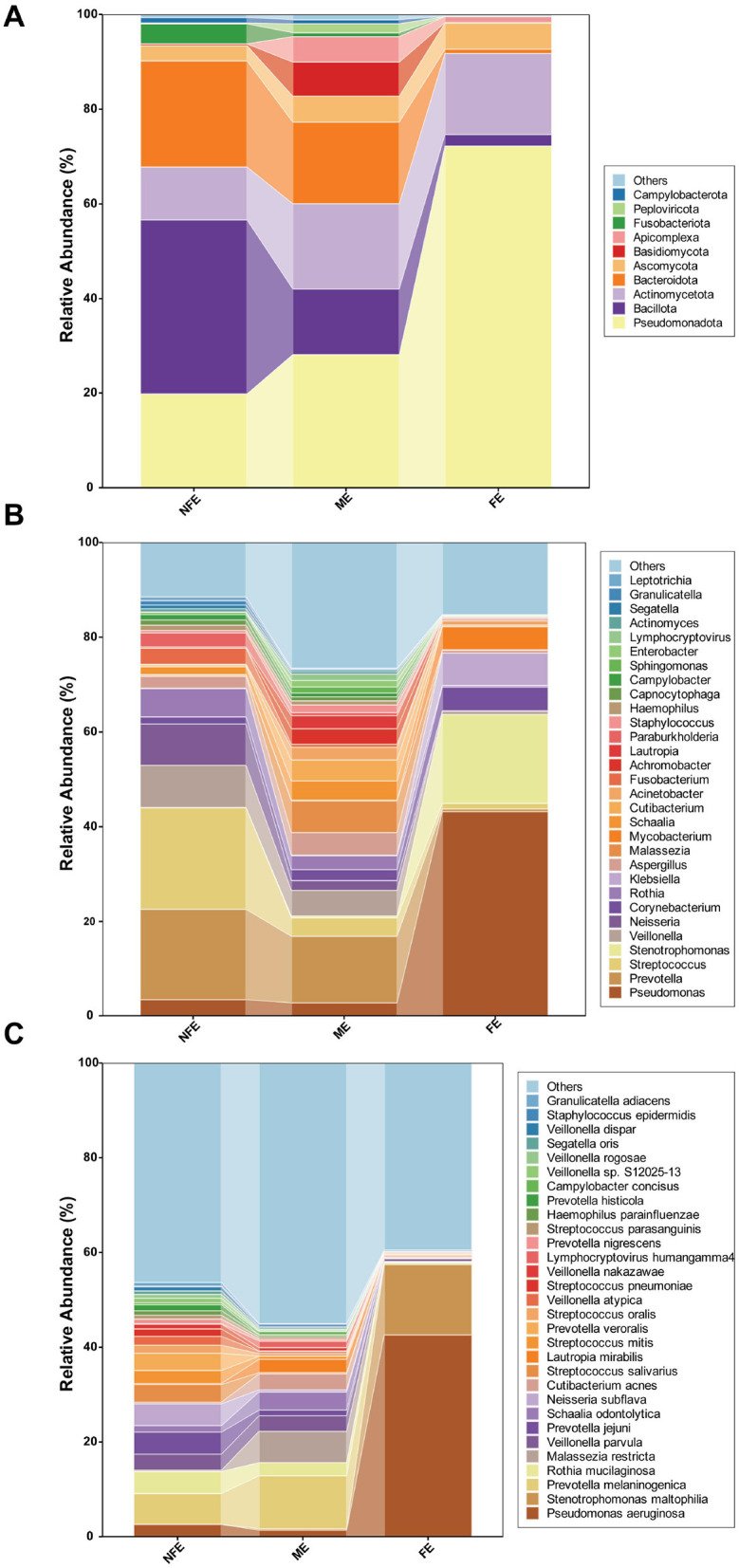
Microbial community composition of bronchoalveolar lavage fluid (BALF) samples. Stacked bar plots show the relative abundance of microbial taxa at the **(A)** phylum, **(B)** genus, and **(C)** species levels across the FE, ME, and NFE groups. Panel **(A)** displays the top 10 most abundant phyla, while panels **(B, C)** illustrate the top 30 most abundant genera and species, respectively.

Deep metagenome sequencing of BALF samples revealed distinct microbial community structures among the FE, ME, and NFE groups at both phylum and genus/species levels (Figures 1A–C). Notably, the NFE and ME groups exhibited comparable profiles dominated by *Pseudomonadota* (formerly *Proteobacteria*; mean relative abundance: 42.82% across all samples), *Bacillota* (15.98%, formerly Firmicutes), *Actinomycetota* (15.78%, formerly known as *Actinobacteria*), and *Bacteroidota* (12.43%, formerly known as *Bacteroidetes*) (Figure 1A). *Pseudomonadota* were prevalent in the FE group, with 72.25% of the relative abundance, whereas in the NFE and ME groups, their relative abundance was 19.79% and 28.08%, respectively. On the other hand, *Bacillota* were more abundant in the NFE and ME groups, with 36.80% and 13.95% of the relative abundance, respectively, compared to the FE group (2.38%). The top 15 bacterial genera included *Pseudomonas, Prevotella, Streptococcus, Stenotrophomonas, Neisseria, Rothia, Veillonella*, and *Corynebacterium* (among others). *Streptococcus, Prevotella*, and *Pseudomonas* were identified as the dominant genera in the NFE, ME, and FE groups, respectively (Figure 1B). Species-level analysis identified *Pseudomonas aeruginosa, Stenotrophomonas maltophilia, Prevotella melaninogenica*, and *Rothia mucilaginosa* as the most abundant species, with *Prevotella melaninogenica* dominating the NFE and ME groups and *Pseudomonas aeruginosa* predominating in the FE group (Figure 1C).

### 3.3 Diversity comparison across exacerbation groups

**Figure 2:**
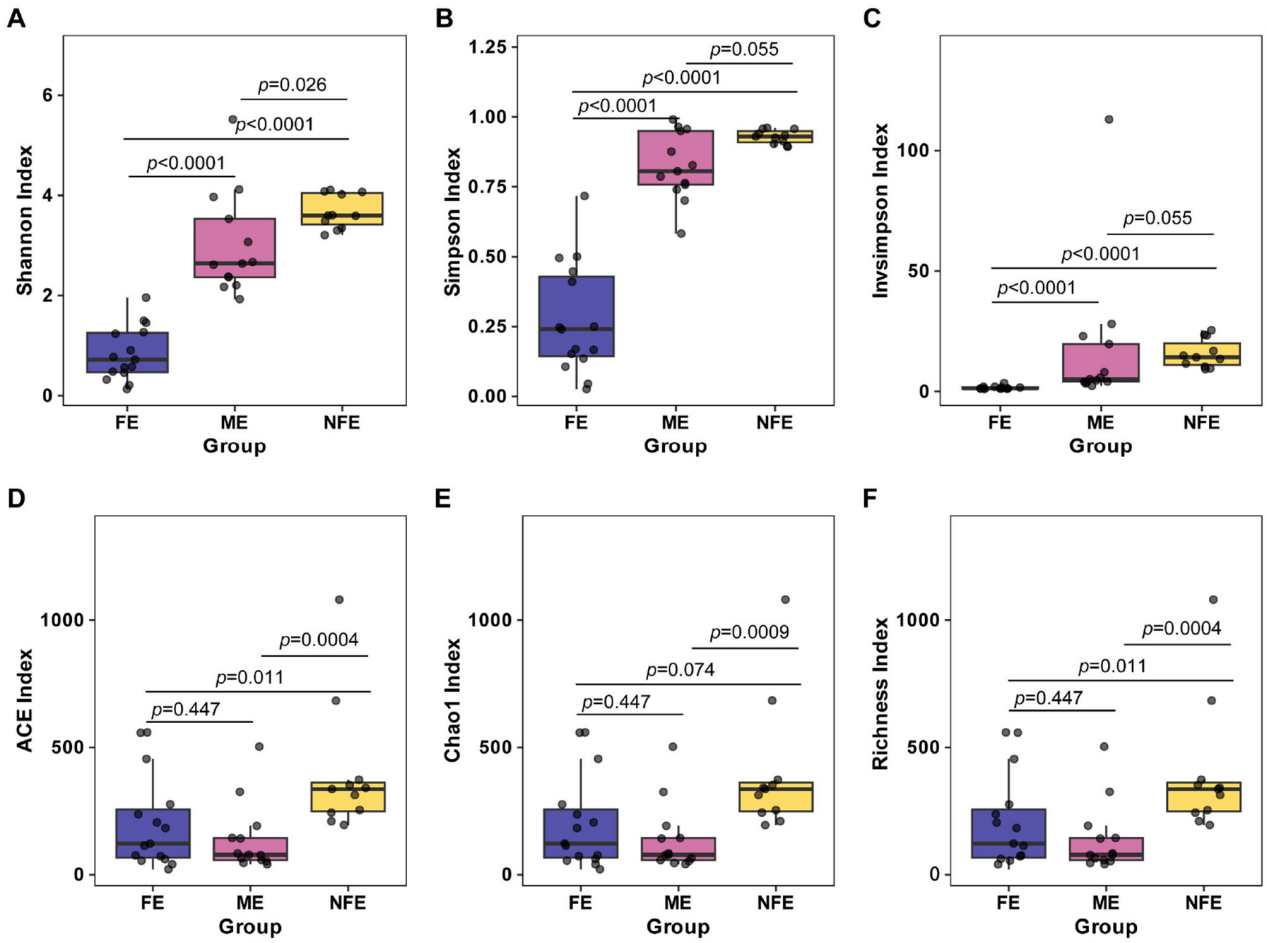
Alpha diversity indices across exacerbation frequency groups. Boxplots **(A–F)** display the distribution of **(A)** Shannon, **(B)** Simpson, **(C)** InvSimpson, **(D)** ACE, **(E)** Chao1, and **(F)** Richness indices among NFE, ME, and FE groups. Statistical comparisons were performed using the Kruskal-Wallis test with Dunn's *post-hoc* test (Bonferroni-adjusted), and exact *p*-values are annotated on the figure.

Alpha diversity, reflecting microbial richness and evenness within individual samples, was significantly higher in the NFE group compared to the FE group at the species level. Specifically, the NFE group exhibited elevated Shannon index (*p* < 0.0001), Simpson index (*p* < 0.0001), Invsimpson index (*p* < 0.0001), ACE index (*p* = 0.011), and Richness index (*p* = 0.011). In contrast, the Chao1 index showed no significant difference between the two groups (*p* = 0.074) (Figure 2).

**Figure 3:**
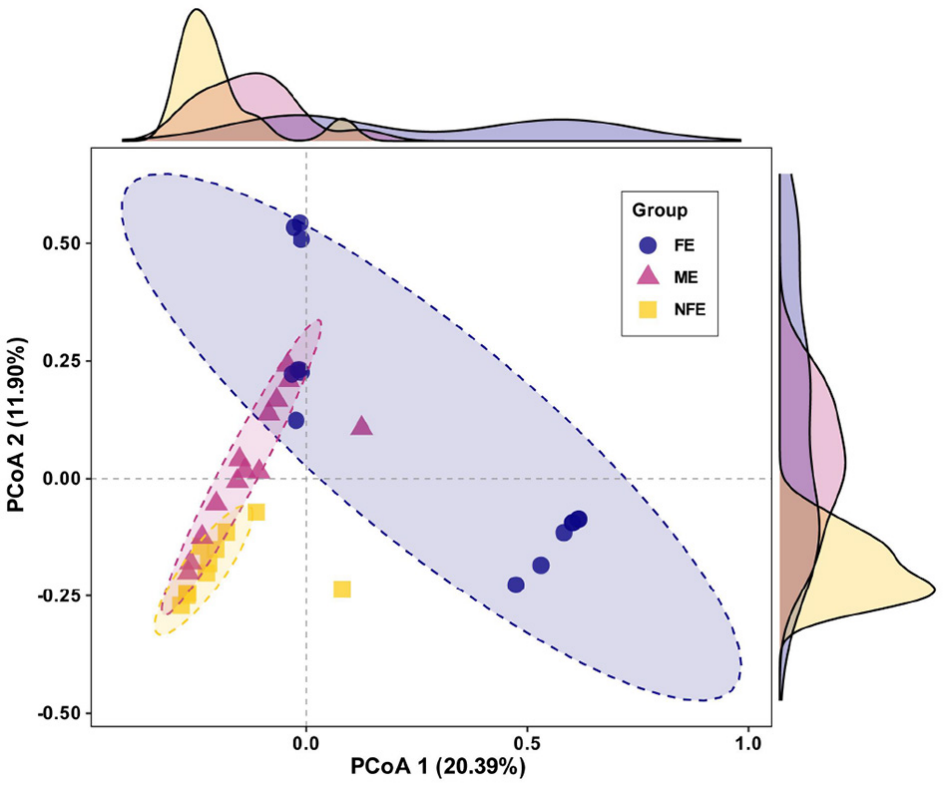
Microbial beta diversity across exacerbation frequency groups. Principal coordinate analysis (PCoA) plot based on Bray-Curtis dissimilarity visualizes the observed clustering pattern of microbial communities among NFE, ME, and FE groups (PERMANOVA, R^2^ = 0.19, *p* = 0.001).

Beta diversity, which assesses differences in microbial community structure across groups, was analyzed using principal coordinate analysis (PCoA) based on Bray–Curtis. The first two principal components accounted for 32.29% of variance explained, with clear clustering observed among the groups. PERMANOVA confirmed significant separation in microbial community composition (*p* = 0.001), supporting distinct structural patterns between groups (Figure 3).

### 3.4 Differential taxa and shifts along the exacerbation spectrum

**Figure 4:**
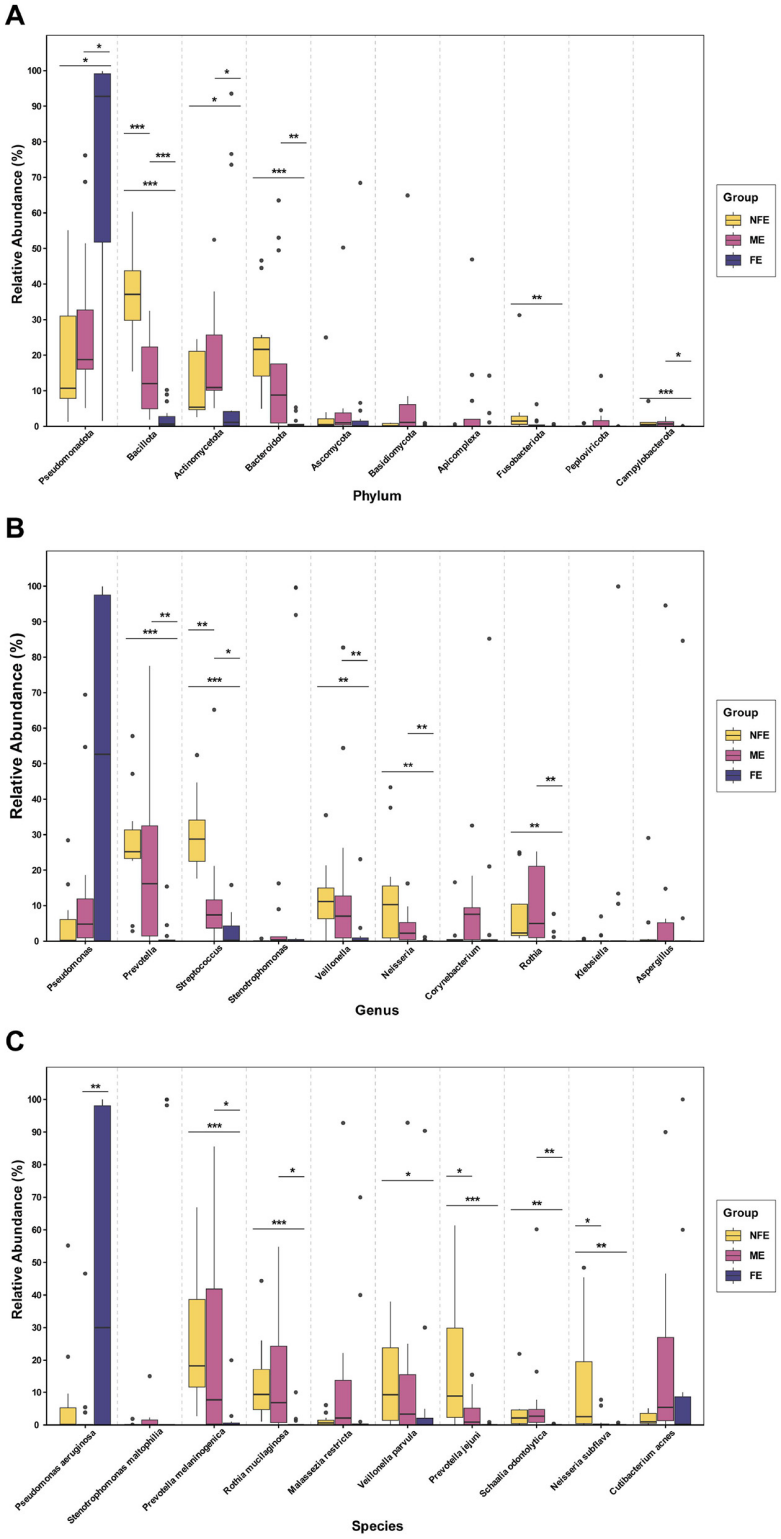
Differential abundance analysis of microbial taxa. Boxplots show the distribution and relative abundance of the top 10 differentially abundant taxa at the **(A)** phylum, **(B)** genus, and **(C)** species levels. Significance was determined using the Kruskal–Wallis test followed by Dunn's *post-hoc* test for multiple comparisons. *P*-values were adjusted using the Benjamini–Hochberg (FDR) method. Significance levels are indicated by asterisks: *p < 0.05, **p < 0.01, ***p < 0.001.

Comparative analysis of the top 10 microbial taxa revealed distinct compositional patterns across the groups (Figure 4). The relative abundance of *Pseudomonadota* was significantly higher in the FE group than in both the NFE and ME groups (*p* = 0.013 for both). In contrast, *Actinomycetota, Bacillota, Bacteroidota, Fusobacteriota*, and *Campylobacterota* were preferentially enriched in the NFE group (all *p* < 0.05). The ME group exhibited a transitional microbiome profile, with intermediate abundances of several phyla, including *Pseudomonadota, Bacillota, Bacteroidota*, and *Fusobacteriota* (Figure 4A). At the genus level, taxa such as *Prevotella, Streptococcus, Neisseria, Veillonella*, and *Rothia* were significantly more abundant in the NFE group compared to the FE group (all *p* < 0.05) (Figure 4B). Similarly, at the species level, *Prevotella melaninogenica, Rothia mucilaginosa, Veillonella parvula, Neisseria subflava, Prevotella jejuni*, and *Schaalia odontolytica* were significantly enriched in the NFE group (all *p* < 0.05). Although *Pseudomonas aeruginosa* was more abundant in the FE group, this difference was not statistically significant (FE vs. NFE: *p* = 0.240) (Figure 4C).

To quantitatively assess whether these microbial changes followed an ordered pattern, we performed Jonckheere–Terpstra trend tests on all taxa that showed significant overall differences in the Kruskal-Wallis test (*p* < 0.05). A significant increasing trend from NFE to ME to FE was confirmed for *Pseudomonadota* (J–T = 3.346, *p* = 0.001). Conversely, significant decreasing trends were identified for the Shannon diversity index (J–T = −5.620, *p* < 0.0001) and for a range of commensal-rich taxa. At the phylum level, these included *Bacillota* (J–T = −5.904, *p* < 0.0001), *Bacteroidota* (J–T = −4.716, *p* < 0.0001), *Fusobacteriota* (J–T = −3.711, *p* < 0.0001), and *Campylobacterota* (J–T = −3.506, *p* < 0.0001). This graded pattern was also observed at the genus level for *Streptococcus* (J–T = −5.367, *p* < 0.0001), *Prevotella* (J–T = −4.355, *p* < 0.0001), *Neisseria* (J–T = −3.983, *p* < 0.0001), *Veillonella* (J–T = −3.771, *p* < 0.0001), and *Rothia* (J–T = −4.222, *p* < 0.0001), and at the species level for *Prevotella melaninogenica* (J–T = −4.273, *p* < 0.0001), *Rothia mucilaginosa* (J–T = −3.856, *p* < 0.0001), *Veillonella parvula* (J–T = −2.831, *p* = 0.005), *Neisseria subflava* (J–T = −3.307, *p* = 0.001), *Prevotella jejuni* (J–T = −4.124, *p* < 0.0001), and *Schaalia odontolytica* (J–T = −2.910, *p* = 0.004). The convergence of significant inter-group differences and robust monotonic trends across taxonomic levels is consistent with a gradient-like reorganization of the lower respiratory microbiome along the exacerbation frequency spectrum.

**Figure 5:**
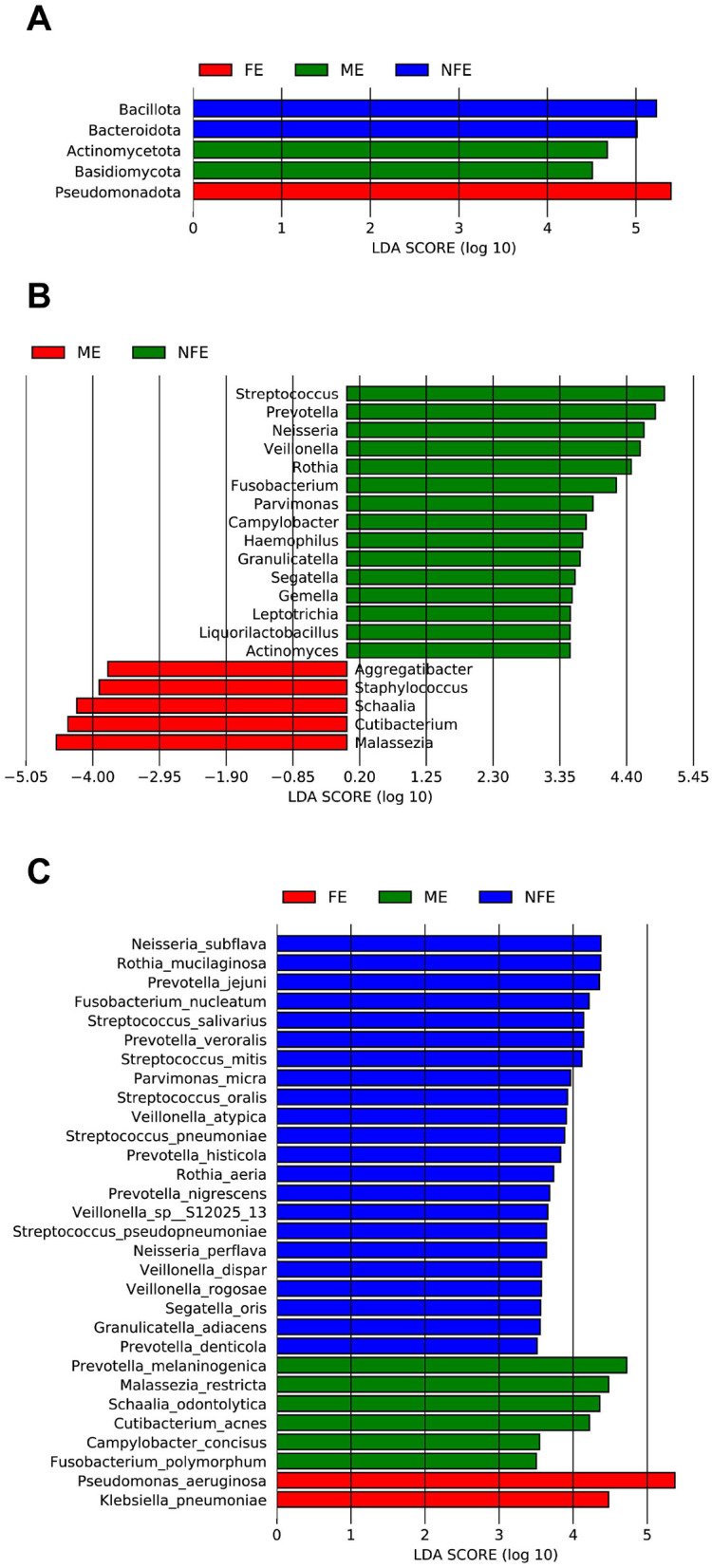
Linear discriminant analysis (LDA) of differentially enriched microbial taxa across the NFE, ME, and FE groups at the **(A)** phylum, **(B)** genus, and **(C)** species levels.

LEfSe analysis further identified specific microbial taxa whose abundances were statistically different among groups (LDA score [log10] > 3.5) (Figure 5). The FE group was characterized by *Pseudomonadota* and *Pseudomonas aeruginosa*. The NFE group was associated with *Bacteroidota, Bacillota, Fusobacteriota*, and genera including *Streptococcus, Prevotella*, and *Neisseria*. The ME group featured a distinct signature, including *Actinomycetota* and the genus *Malassezia*.

### 3.5 Correlation between the microbiome and clinical indexes

**Figure 6:**
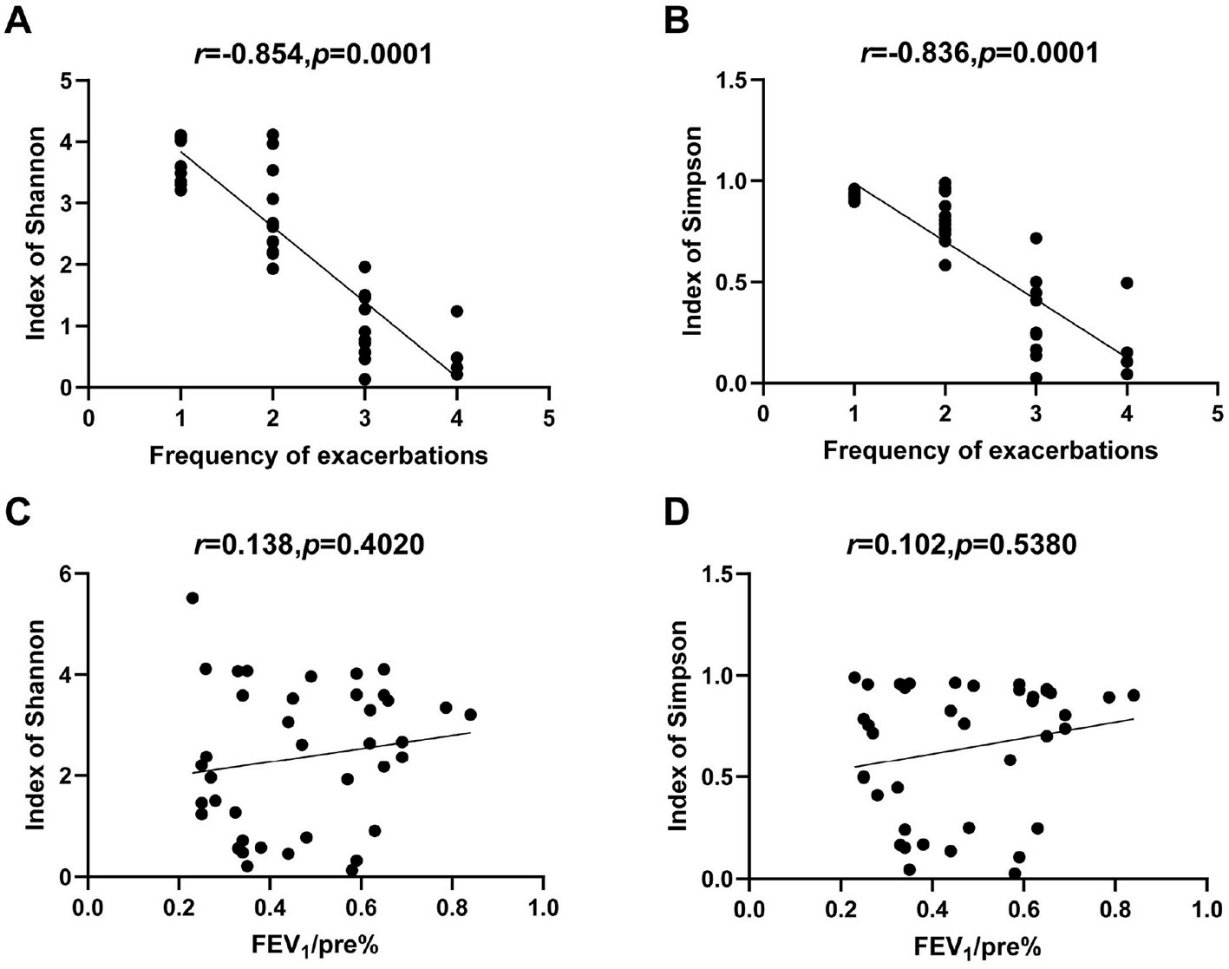
Correlations between microbial alpha diversity and clinical indexes. **(A)** Correlation of acute exacerbation frequency with Shannon index. **(B)** Correlation of acute exacerbation frequency with Simpson index. **(C)** Correlation of FEV_1_/predicted (%) with Shannon index. **(D)** Correlation of FEV_1_/predicted (%) with Simpson index.

Spearman correlation analysis was performed to assess the relationships between clinical variables and microbiome diversity index. The frequency of exacerbation was strongly negatively correlated with both the Shannon index (*r* = −0.842, *p* < 0.0001) and Simpson index (*r* = −0.836, *p* < 0.0001). In contrast, FEV_1_/pre (%) and FEV_1_/FVC (%) showed no significant correlation with either Shannon or Simpson index (Figure 6). The neutrophil-to-lymphocyte ratio (NLR) and COPD duration were also found to be negatively correlated with the Shannon and Simpson index (Supplementary Figure 1).

**Figure 7:**
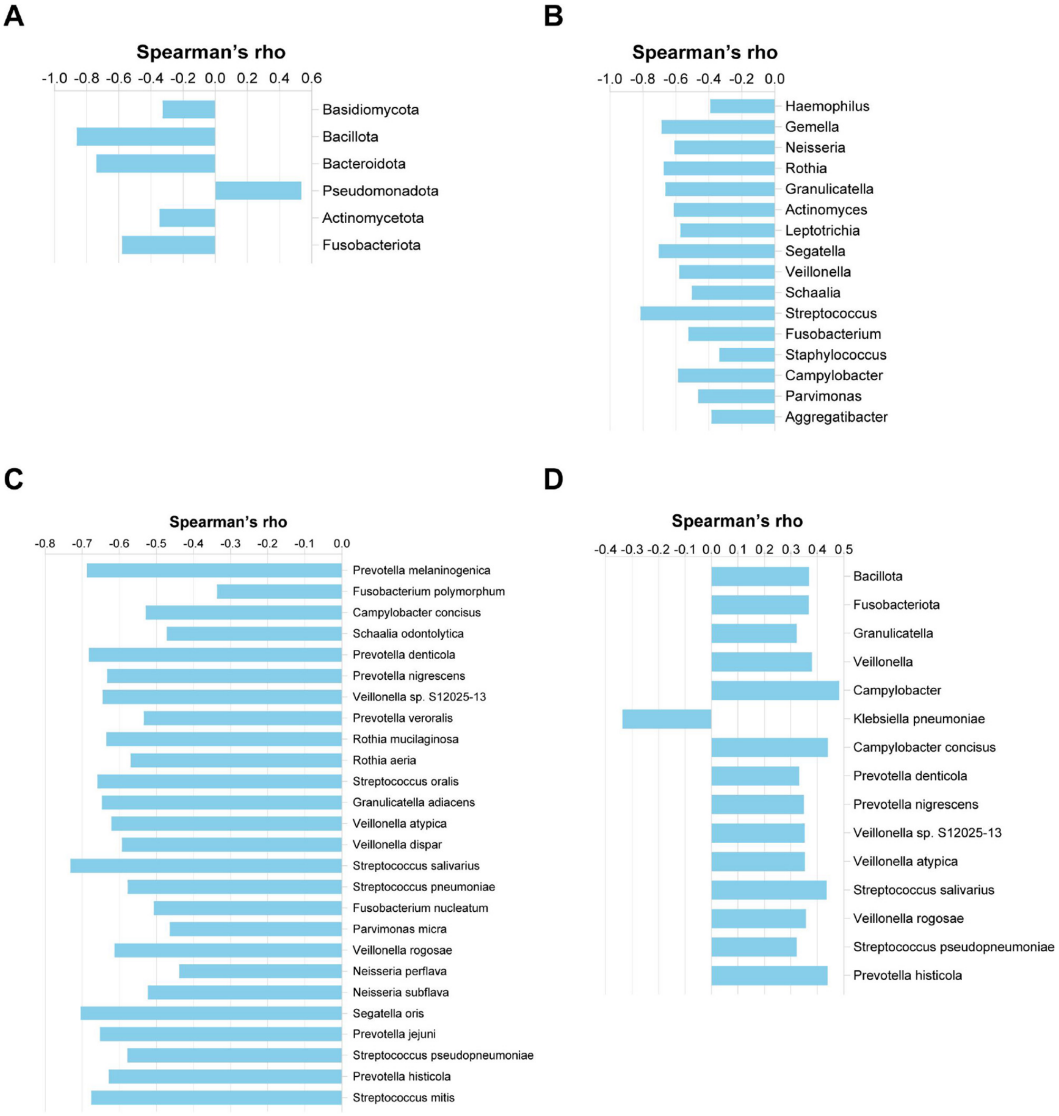
Correlations between microbial relative abundance and clinical indexes. **(A)** Phylum-level correlation with acute exacerbation frequency. **(B)** Genus-level correlation with acute exacerbation frequency. **(C)** Species-level correlation with acute exacerbation frequency. **(D)** Correlation of microbial relative abundance with FEV_1_/predicted (%) at the phylum, genus, and species levels.

We further examined the associations between microbial taxa (identified by LDA) and clinical parameters. The relative abundance of *Pseudomonadota* was positively correlated with exacerbation frequency (*r* = 0.536, *p* < 0.0001), whereas *Fusobacteriota, Actinomycetota, Bacteroidota*, and *Bacillota* showed significant negative correlations (*r* = −0.58, −0.347, −0.74, and −0.862, respectively; all *p* < 0.05) (Figure 7A). A total of 43 microbial genera and species were negatively correlated with exacerbation frequency (*r* ranged from −0.814 to −0.336), 60.5% (26/43) of which belonged to the *Bacillota* and *Bacteroidota* phyla. Notably, *Streptococcus* exhibited a strong negative correlation (*r* = −0.814, *p* < 0.0001) (Figures 7B, C). Exacerbation frequency was also negatively associated with both FEV_1_/pre (*r* = −0.434, *p* = 0.006) and FEV_1_/FVC (*r* = −0.404, *p* = 0.011) (Supplementary Figure 2). *Klebsiella pneumoniae* proved to have a negative correlation with FEV_1_/pre(*r* = −0.336, *p* = 0.036). A total of 12 microbial genera and species (r ranged from 0.322 to 0.483) showed positive correlations with FEV_1_/pre and negative correlations with acute exacerbation frequency, 83.3% (10/12) of which belonged to phylum *Bacillota* and *Bacteroidota* (Figure 7D).

**Figure 8:**
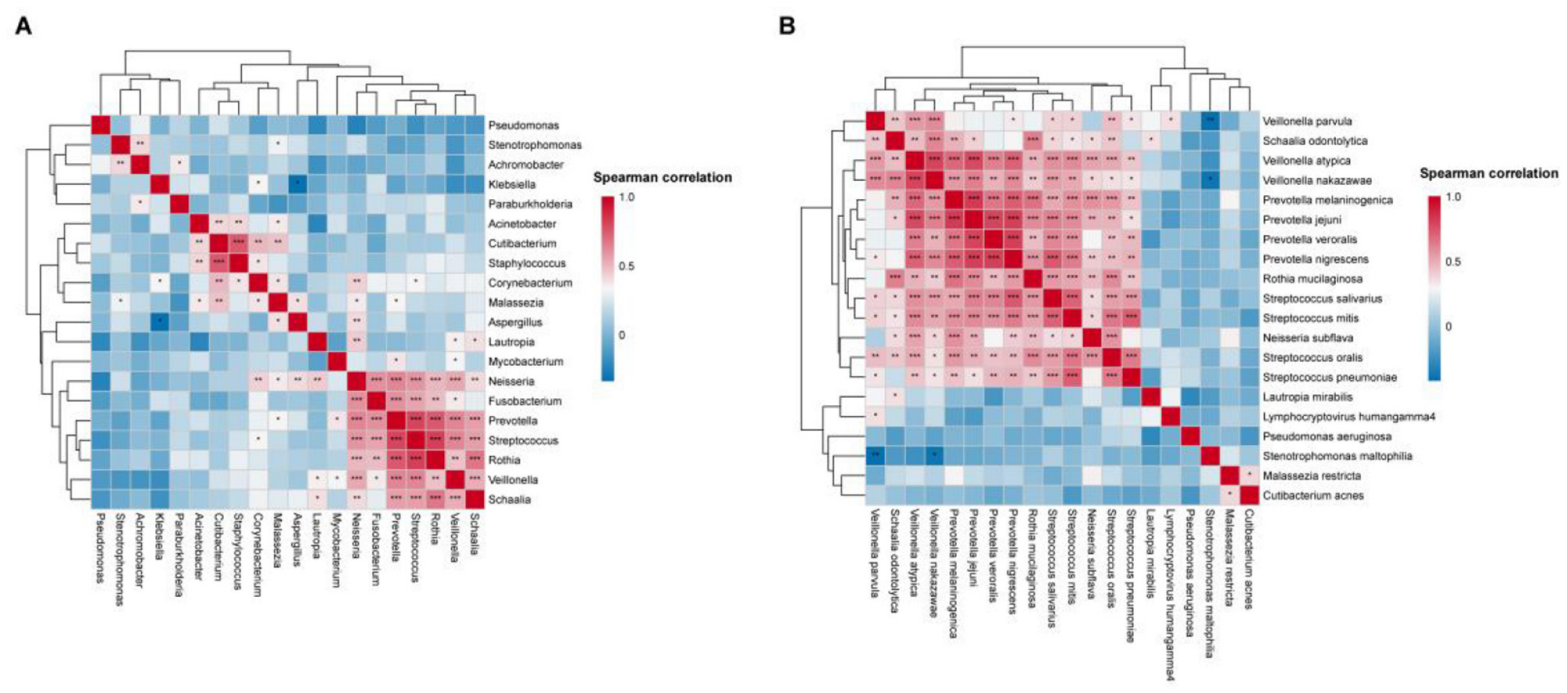
BALF microbiome networks separately in AECOPD patients. Heatmaps display Spearman correlation coefficients among the relative abundances of the top 20 **(A)** genera and **(B)** species. Significant correlations (*p*-value < 0.05) are marked with an asterisk.

To explore potential co-occurrence and co-exclusion relationships within the microbial community, spearman correlation analysis among the top 20 microbial genera and species was performed (Figure 8).

## 4 Discussion

To the best of our knowledge, this study represents the first application of metagenomic next-generation sequencing (mNGS) to systematically characterize the bronchoalveolar lavage fluid (BALF) microbiome in AECOPD patients stratified by exacerbation frequency. Our principal findings reveal a significant microbial gradient across the exacerbation spectrum (from NFE to ME to FE), characterized by a stepwise expansion of *Pseudomonadota* and a concomitant decline in commensal-rich taxa, accompanied by a marked loss of microbial alpha diversity. Correlation analyses further support the potential link between these microbial shifts and key clinical phenotypes, such as exacerbation frequency and lung function.

### 4.1 Microbial diversity decreases along the exacerbation spectrum

Our metagenomic analysis of bronchoalveolar lavage fluid (BALF) revealed a significant and progressive decline in microbial alpha diversity in the lower airways with increasing exacerbation frequency (Shannon index, Jonckheere–Terpstra test statistic = −5.620, *p* < 0.0001). This diversity gradient may reflect an impairment of “colonization resistance”—an ecological concept wherein a diverse commensal microbiota typically prevents pathogen expansion through direct mechanisms such as niche competition, metabolic exclusion, and production of antimicrobial substances (Caballero-Flores et al., 2023). We observed that in frequent exacerbators, the expansion of potential pathogens coexists with a reduction in complex commensal communities, which may represent a manifestation of pulmonary microecological destabilization that is associated with the exacerbation-prone phenotype. This pattern of diversity loss observed in the lower respiratory tract is consistent with previous studies based on upper airway and sputum samples. For instance, Pragman et al. similarly reported reduced alpha-diversity in the oropharyngeal and sputum microbiota of COPD frequent exacerbators (Pragman et al., 2019). Furthermore, the association between microbial community simplification and adverse clinical outcomes has been demonstrated in multiple studies: Galiana et al. found that sputum microbial diversity was lower in patients with severe COPD compared to those with moderate disease (Galiana et al., 2014), while Leitao Filho et al. further established that reduced microbial diversity was independently associated with an increased one-year mortality risk in COPD patients (Leitao Filho et al., 2019). Collectively, these findings suggest that microbial diversity holds promise as an important biomarker for identifying high-risk patients and guiding future microbiome-targeted intervention strategies.

### 4.2 Microbial composition shows an ordered shift along the exacerbation spectrum

A core finding of our study is the profound and ordered gradient shift in microbial community structure along the exacerbation spectrum. Jonckheere–Terpstra trend tests confirmed a significant stepwise increase in the relative abundance of *Pseudomonadota* from the NFE to the ME to the FE group. This finding is consistent with a prospective cohort study demonstrating that the abundance of *Pseudomonadota* (formerly *Proteobacteria*) was independently associated with acute exacerbation events and frequency (Wang et al., 2016). Similarly, Wang et al. also reported an increase in *Pseudomonadota* and a decrease in *Bacillota* (formerly *Firmicutes*) during exacerbations (Wang et al., 2019).

Conversely, several commensal-rich phyla, including *Bacillota, Bacteroidota*, and *Fusobacteriota*, showed a significant decreasing trend with increasing exacerbation frequency. This graded pattern was more pronounced at the genus and species levels, with significant declines in taxa such as *Streptococcus, Prevotella, Veillonella, Rothia, Prevotella melaninogenica*, and *Rothia mucilaginosa*. These microorganisms are considered core members of the healthy or stable COPD lung microbiome (Pragman et al., 2018; Faner et al., 2017). Studies have shown that the abundance of *Prevotella* increases with the alleviation of airflow limitation (Su et al., 2022), while Rothia is more abundant in mild COPD and may exert anti-inflammatory effects by suppressing the NF-κB pathway (Rigauts et al., 2022). Importantly, this specific microbiota configuration—characterized by the loss of commensals like *Veillonella* and enrichment of pathogens like *Staphylococcus*—has been independently established as a significant risk factor for increased mortality in AECOPD patients (Leitao Filho et al., 2019).

### 4.3 Unique mycobiota signature and its potential implications in the ME Group

An intriguing finding from our LEfSe analysis was the specific enrichment of the fungal genus *Malassezia* in the ME group. While our study primarily focused on bacteria and the low biomass for fungi limits definitive conclusions, this signal suggests a potential, underappreciated role for the mycobiome in COPD progression. Previous research on the COPD lung mycobiome has predominantly focused on *Candida* and *Aspergillus*, leaving the ecological significance of *Malassezia* in the respiratory tract unclear (Nguyen et al., 2015; de Dios Caballero et al., 2022; Garaci et al., 2024). Its co-occurrence with taxa like *Prevotella melaninogenica* in the ME group may represent a unique transitional ecological niche during the exacerbation process. This hypothesis-generating discovery warrants validation in future studies specifically designed with sufficient power to characterize the lung mycobiome.

### 4.4 Correlation between microbiome and clinical phenotypes

We identified robust associations between microbiome shifts and key clinical indices. Exacerbation frequency was strongly and negatively correlated with lung function parameters (FEV_1_/pred and FEV_1_/FVC), underscoring its role as a key marker of disease progression. Furthermore, the microbiome was closely linked to clinical phenotypes: exacerbation frequency correlated positively with the abundance of *Pseudomonadota* and negatively with the abundance of multiple commensal-rich phyla/genera/species. Notably, we identified a set of microorganisms (e.g., *Streptococcus*) that were negatively correlated with exacerbation frequency and positively correlated with FEV_1_/pred. This observation aligns with the perspective of the latest GOLD report, which states that dysbiosis is associated with the presence and characteristics of COPD, such as exacerbation frequency, potentially by altering mucosal defense and stimulating lung inflammation [Global Initiative for Chronic Obstructive Lung Disease (GOLD), 2024]. This further highlights the clinical relevance of the identified microbial changes, placing them within the clinical continuum of lung function impairment.

However, it must be emphasized that the cross-sectional design of this study precludes causal inference. Frequent exacerbations and poor lung function could be either a cause or a consequence of microbial dysbiosis, and both may be driven by underlying host immune factors (Plichta et al., 2019; Wilde et al., 2024). For instance, ecological dysbiosis of the lung microbiome has been implicated in the pathophysiology of chronic obstructive pulmonary disease (COPD) and other respiratory conditions through its role in modulating host immune responses. Furthermore, the complex interplay between the pulmonary microbiome and the host environment underscores these relationships, with tissue-associated microbial communities potentially participating more directly in this dynamic process (Sze et al., 2012; Yi et al., 2022).

### 4.5 Study strengths, limitations, and future perspectives

A key strength of this study is the use of BALF, which more accurately reflects the lower respiratory tract milieu, combined with high-resolution metagenomic next-generation sequencing (mNGS). This approach not only provided a refined microbial profile at the species level but also serendipitously enabled the detection of unique non-bacterial signals, such as the *Malassezia* signature in the ME group, underscoring the value of an unbiased sequencing method.

This study also has several limitations. First, the relatively small sample size and single-center, cross-sectional design limit the generalizability of our findings and prevent the establishment of causal or temporal relationships. Second, although we excluded patients with recent antibiotic use, the FE group's history of more frequent exacerbations likely resulted in greater cumulative antibiotic exposure, a potential confounding factor that we could not fully quantify or adjust for. Furthermore, our study relied on patient-reported exacerbation frequency as a key stratification variable. While this is a well-established and clinically relevant metric, we lacked comprehensive data on prior hospitalization history for exacerbations. Therefore, it is possible that exacerbation frequency alone may not fully capture the cumulative burden of severe exacerbation events requiring hospitalization. Additionally, the analysis of non-bacterial domains (e.g., fungi) was inherently limited by statistical power issues due to their low biomass. Consequently, we prioritized the robust bacterial community analysis while explicitly framing the fungal signals as hypothesis-generating discoveries for future validation. Finally, the substantial interpersonal variation in microbiome composition necessitates validation of our findings in larger, prospective cohorts.

Based on these considerations, we propose that future research should: (1) expand to multi-center, longitudinal cohorts to validate and extend our model of microbial succession along the exacerbation spectrum; and (2) employ domain-specific techniques (e.g., ITS sequencing) to definitively characterize the role of the mycobiota and its interactions with the bacterial community.

## 5 Conclusion

Collectively, our findings delineate a gradient of airway microbial ecology along the exacerbation frequency spectrum. We propose that the observed expansion of *Pseudomonadota*, coupled with the depletion of commensal *Bacillota* and *Bacteroidota*, may collectively is associated with to the exacerbation-susceptible state in COPD. Future studies are needed to test if this dysbiotic configuration actively drives poor clinical outcomes.

## Data Availability

The datasets presented in this article are not readily available because China's Regulations on the Management of Human Genetic Resources prohibit public deposition of raw metagenomic data containing human genetic information. Requests to access the datasets (under a Data Use Agreement and in compliance with applicable regulations) should be directed to the corresponding author.
