# Supplementary figures and images for: Tripartite exacerbation stratification in AECOPD suggests a gradient of lower airway dysbiosis: a metagenomic transition from commensal taxa to pseudomonadota dominance
Source: Front Microbiol. 2025 Nov 24;16:1588029. doi: 10.3389/fmicb.2025.1588029 (PMC12682771; doi:10.3389/fmicb.2025.1588029)

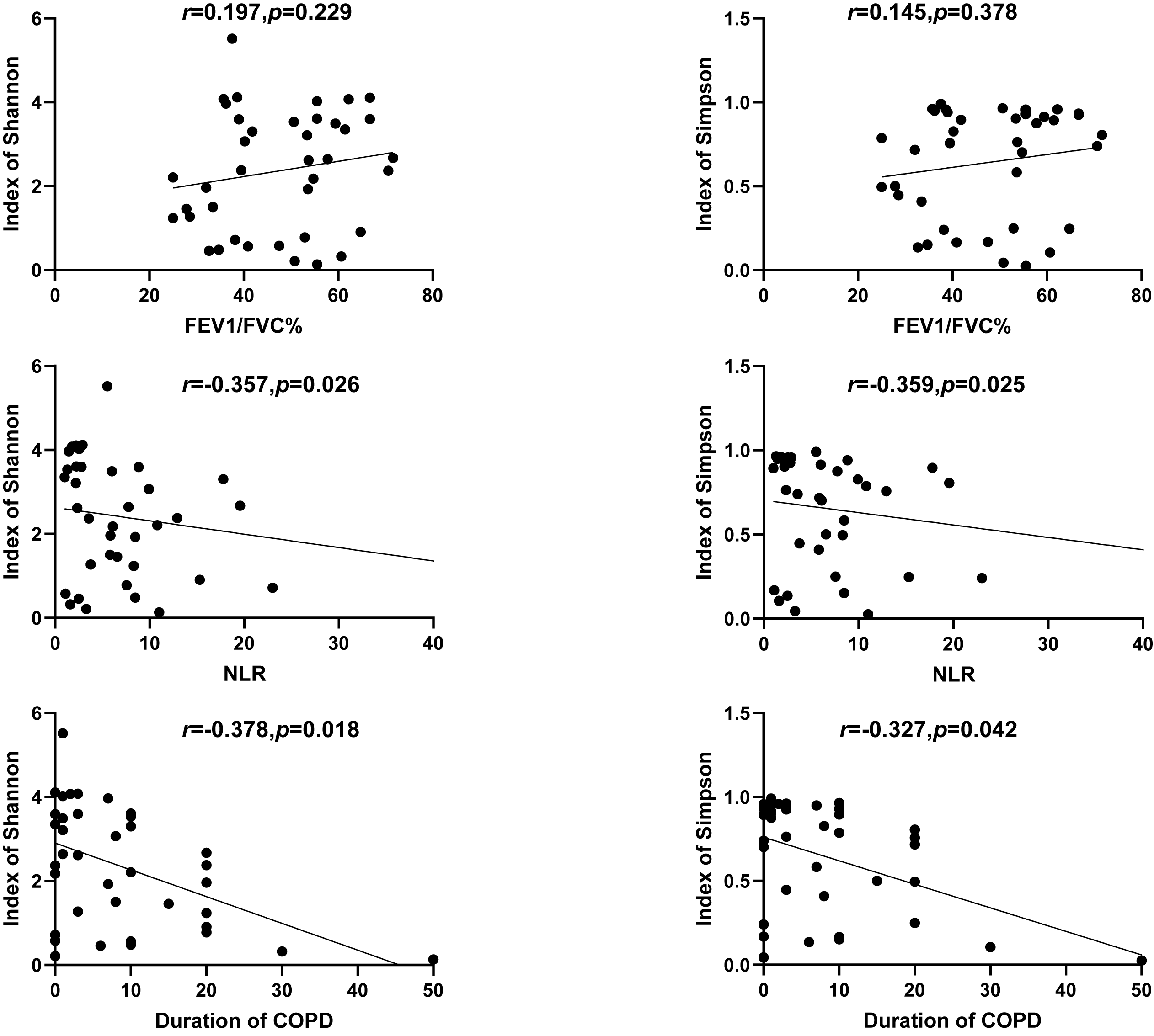

Supplement: Supplementary file 5 [file Image_1.tif]

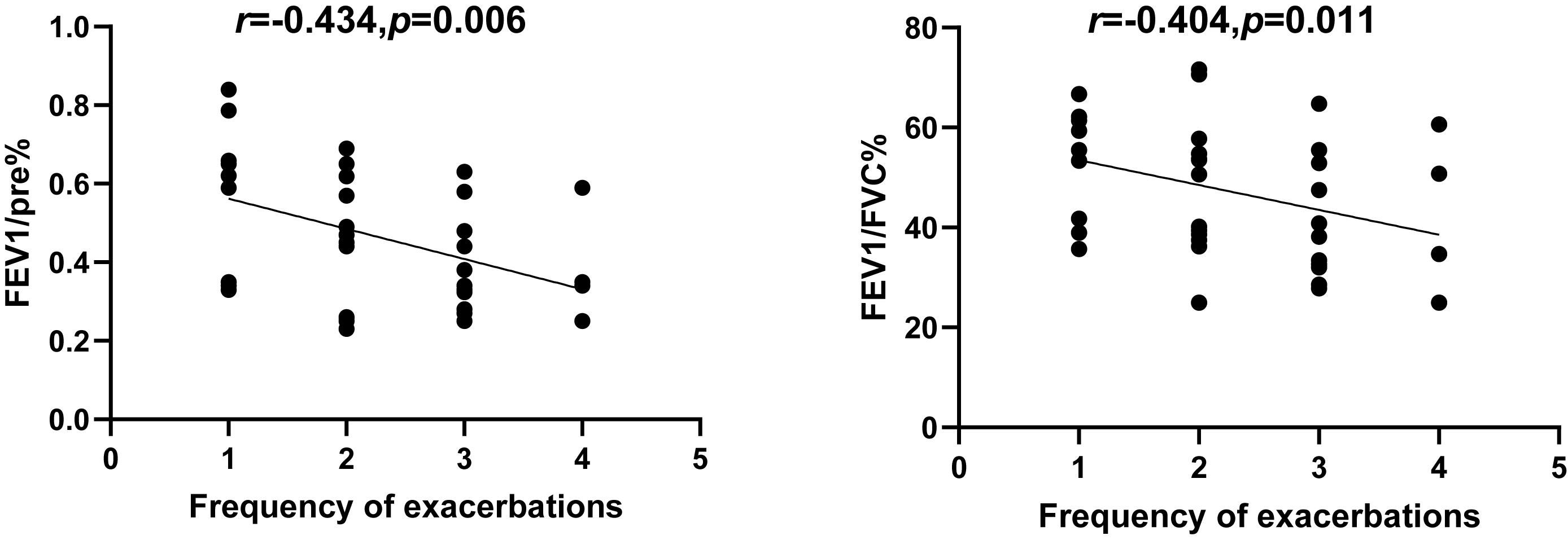

Supplement: Supplementary file 6 [file Image_2.tif]
